# Adding aprepitant to palonosetron does not decrease carboplatin-induced nausea and vomiting in patients with gynecologic cancer

**DOI:** 10.1186/s40780-021-00204-z

**Published:** 2021-06-01

**Authors:** Yuko Watanabe, Yoshitaka Saito, Takashi Mitamura, Yoh Takekuma, Mitsuru Sugawara

**Affiliations:** 1 Department of Pharmacy, Hokkaido University Hospital, Kita 14-jo, Nishi 5-chome, Kita-ku, Sapporo, 060-8648 Japan; 2 Department of Obstetrics and Gynecology, Faculty of Medicine and Graduate School of Medicine, Hokkaido University, Kita 15-jo, Nishi 7-chome, Kita-ku, Sapporo, Hokkaido 060-8648 Japan; 3 Laboratory of Pharmacokinetics, Faculty of Pharmaceutical Sciences, Hokkaido University, Kita 12-jo, Nishi 6-chome, Kita-ku, Sapporo, 060-0812 Japan

**Keywords:** Aprepitant, Carboplatin, TC, Nausea, CINV, MEC

## Abstract

**Background:**

Recently, aprepitant has been recommended in carboplatin-based regimens, but there are limited reports on the efficacy of administering aprepitant, palonosetron, and dexamethasone (DEX) in carboplatin-containing regimens. Moreover, because aprepitant is an expensive drug, confirming its effectiveness is very important from the medical cost perspective. In this study, we examined the efficacy of prophylactically administered aprepitant, palonosetron and DEX, in paclitaxel and carboplatin (TC) combination chemotherapy.

**Methods:**

Patients with gynecologic cancer who were treated with paclitaxel (175 mg/m^2^) and carboplatin (area under the curve, AUC = 5–6) combination chemotherapy were retrospectively evaluated. The complete response (CR) rate, severity of nausea, and incidence of anorexia in the first course were compared between patients who did not receive aprepitant (control group) and those who received (aprepitant group).

**Results:**

The 106 patients were divided into two groups, consisting of 52 and 54 the control and aprepitant groups, respectively, and the patient background showed no significant difference between both groups. The CR rate of the overall phase between the control and aprepitant groups was 73.1 vs. 74.1%, that in the acute phase was 98.1 vs. 100%, and in the delayed phase was 75.0 vs. 74.1%, respectively, without any significant difference. The severity of nausea and incidence of anorexia were also not significantly different between both groups.

**Conclusions:**

The results of the study suggest that adding aprepitant to palonosetron and DEX does not prevent carboplatin-induced nausea and vomiting in gynecologic cancer patients. Therefore, adding aprepitant to palonosetron does not decrease carboplatin-induced nausea and vomiting in patients with gynecologic cancer.

## Introduction

Paclitaxel and Carboplatin (TC) combination chemotherapy is regarded as the standard regimen for gynecologic cancer and, therefore, is administered to most of these patients [1–4].

Chemotherapy-induced nausea and vomiting (CINV) is one of the most frequent adverse effects, and uncontrolled CINV may limit the dose intensity of chemotherapy and decrease the patient’s quality of life [5–7]. Carboplatin-containing therapies such as TC have been classified as moderate emetogenic chemotherapy (MEC). Two-drug combinations consisting of a 5-hydroxytrypatmine-3 receptor antagonist (5-HT_3_RA) and dexamethasone (DEX) have been recommended with the option of adding aprepitant, which is a neurokinin-1 receptor antagonist (NK_1_RA) [8, 9].

However, recent guidelines for antiemetic treatments published by the Multinational Association of Supportive Care in Cancer (MASCC), European Society of Medical Oncology (ESMO), American Society of Clinical Oncology (ASCO), National Comprehensive Cancer Network (NCCN) and the Japanese Society of Clinical Oncology (JSCO) guidelines for CINV have reclassified carboplatin [10–13]. Specifically, the drug has been reported to have the highest CINV risk in patients receiving MEC, and the guidelines suggest the administration of antiemetics according to the recommendations for the highly emetogenic chemotherapy (HEC) classification [10–13]. Aprepitant has been shown to be effective for CINV when added to 5-HT_3_RAs and DEX in HEC and MEC regimens [14–18]. However, previous studies have reported that adding aprepitant to conventional therapies does not prevent CINV during the first carboplatin administration [19–21]. Furthermore, although palonosetron is preferred to first-generation 5-HT_3_RAs such as granisetron or ondansetron for MEC or HEC [22, 23], there are few reports on the efficacy of aprepitant, palonosetron, and DEX in carboplatin-containing regimens [5, 6, 14, 15]. In particular, being a member of the female sex is known to be a risk factor for CINV [18], making the efficacy of this agent in gynecologic cancer patients controversial. Moreover, because aprepitant is an expensive medicine, it is important to confirm its effectiveness from the perspective of the medical cost [24–26].

In this study, we evaluated the efficacy of prophylactic administration of aprepitant, palonosetron, and DEX as part of a carboplatin-based regimen.

## Patients and methods

### Patients

Patients who received the combination chemotherapy regimen of paclitaxel (175 mg/m^2^) and carboplatin (area under the curve; AUC = 5–6) every 3–4 weeks as a first line chemotherapy for gynecologic cancer from January 2015 to June 2019 were enrolled in this retrospective study.

Patients were divided into two groups, consisting of one that did not receive aprepitant from January 2015 to June 2017 (control), and another that did receive aprepitant from July 2017 to June 2019 (aprepitant group). Patients who experienced uncontrolled nausea and vomiting or were regularly administered antiemetic drugs such as metoclopramide, domperidone, lorazepam, prochlorperazine, and patients with insufficient data were excluded. This retrospective study was approved by the Institutional Review Board of the Hokkaido University Hospital (approval number: 018–0390) and conducted in accordance with the Declaration of Helsinki. In view of the retrospective nature of the study, informed consent from the subjects was not mandated.

### Treatment methods

Patients in the control group were treated with palonosetron 0.75 mg and DEX 16.5 mg intravenously on day 1, and DEX 8 mg orally on day 2–3. Patients in the aprepitant group were treated with aprepitant 125 mg orally, palonosetron 0.75 mg, and DEX 16.5 mg intravenously on day 1, followed by aprepitant 80 mg and DEX 4 mg orally on day 2–3. Antiemetic drugs such as metoclopramide domperidone or prochlorperazine were administered as rescue medications according to the physician’s decision.

### Evaluation criteria

All patients were hospitalized, and the required information was obtained from their daily medical records. In the evaluation period, day 1–5, day 1, and day 2–5 were defined as the overall, acute, delayed phases at the first implementation of TC as described previously [27]. The primary endpoint was a complete response (CR), which was defined as the absence of emetic events, vomiting, and need for rescue antiemetic treatment in the overall phase. Secondary endpoints were configured the CR rate in the acute and delayed phase, the severity of nausea, and the prevalence of anorexia. The symptoms of CINV were evaluated based on the real-time assessment of daily physicians and/or pharmacists according to Common Terminology Criteria for Adverse Events version 4.0, when the patients were hospitalized.

### Statistical analysis

The differences in patient background between the control and aprepitant groups were assessed using Fisher’s exact probability test for categorical outcome variables, and the Mann-Whitney *U* test for the continuous parameters. Differences in the CR rate and incidence of anorexia between the two groups were analyzed using Fisher’s exact probability test. Differences in the degree of severity of nausea were assessed using the Mann-Whitney U test. All analyses were carried out using the JMP version 14.0 statistical software (SAS Institute Japan, Tokyo, Japan). A significant difference was accepted for results with a *P*-value < 0.05 for all tests.

## Results

### Patients

**Fig. 1:**
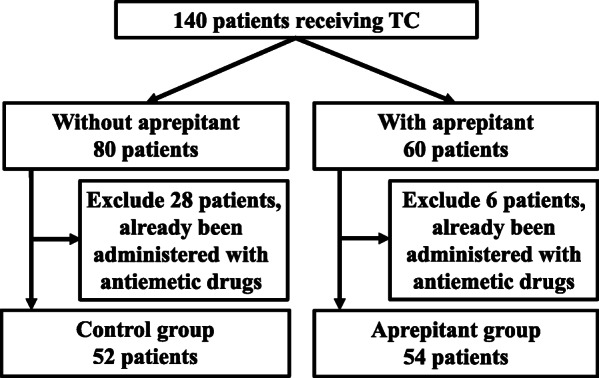
Study flow chart. TC: paclitaxel and carboplatin

A total of 140 patients were enrolled in this study, including 34 who were excluded during screening. Then, 106 patients were divided into two groups, consisting of 52 and 54 patients in the control and aprepitant group, respectively (Fig. 1). Patients’ background information is shown in Table 1. There was no significant difference between the control and aprepitant groups in age, performance status, cancer diagnosis, staging, chemotherapy setting, body surface area, drinking habit, and TC dosage. The proportion of patients who were administered carboplatin at a dose of AUC = 5 was 100% in the control group and 98.1% in the aprepitant group, and that of patients administered at a dose of AUC = 6 was 0 and 1.9% in the control and aprepitant groups, respectively, without significant differences. Patients with renal dysfunction (grade 1 or higher serum creatinine elevation), and those with liver dysfunction (grade 1 or higher aspartate transaminase, alanine aminotransferase, and total bilirubin elevation) were not different between the groups. There were no patients with grade 2 or higher adverse events.

**Table 1:** Patients’ background

	Control group (*n* = 52)	Aprepitant group (*n* = 54)	*P*-value
Age (median, range)	59 (29–83)	58 (38–81)	0.70
Performance status (ECOG) 0–1/2–3	51/1	53/1	1.00
Cancer diagnosis (number, %)			
Ovarian or tubal cancer	17 (32.7)	22 (40.7)	0.43
Uterine cancer	28 (53.8)	28 (51.9)	0.85
Cervical cancer	6 (11.5)	1 (1.9)	0.06
Vaginal cancer	0 (0)	1 (1.9)	1.00
Double cancer	1 (1.9)	2 (3.7)	1.00
Staging (number, %)			
I/II/III	39 (75.0)	39 (72.2)	0.83
IV/recurrence	13 (25.0)	15 (27.8)	
Chemotherapy setting			
neoadjuvant or adjuvant	44 (84.6)	49 (90.7)	0.34
for advanced cancer	8 (15.4)	5 (9.3)	
Height (cm) (median, range)	155.5 (144.6–172.1)	154.2 (142.0–169.5)	0.49
Body weight (kg) (median, range)	54.7 (35.6–84.6)	54.3 (37.7–89.2)	0.79
Body surface area (m^2^) (median, range)	1.53 (1.24–1.85)	1.54 (1.21–1.87)	0.90
Drinking habit (number, %)	24 (46.2)	21 (38.9)	0.57
Carboplatin dosage (AUC) (number, %)			
5	52 (100)	53 (98.1)	1.00
6	0 (0)	1 (1.9)	
Paclitaxel dosage (mg) (median, range)	261.7 (220–320)	266.8 (210–325)	0.43
Dose reduction (number, %)	3 (5.8)	1 (1.9)	0.36
Renal dysfunction (number, %)	4 (7.6)	5 (9.3)	1.00
Liver dysfunction (number, %)	9 (17.3)	12 (22.2)	0.63

Renal dysfunction: grade 1 or higher serum creatinine elevation

Liver dysfunction: grade 1 or higher aspartate transaminase, alanine aminotransferase, and total bilirubin elevation

*AUC* area under the carve

### Comparison of CR rate

**Fig. 2:**
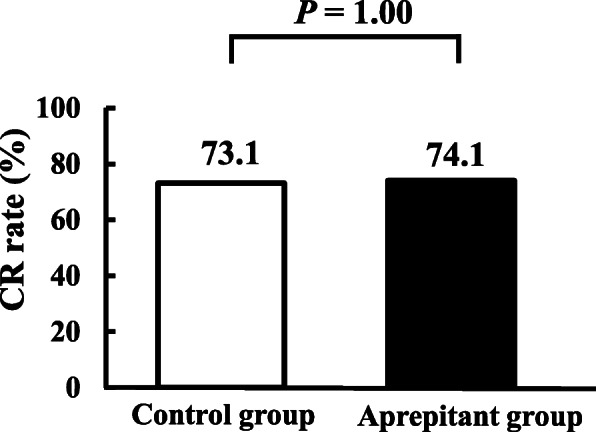
Complete response (CR) rate in overall phase

**Fig. 3:**
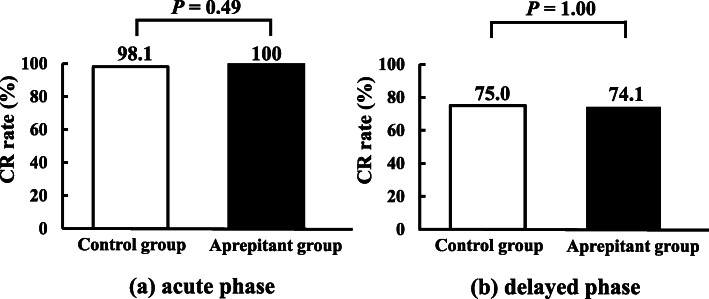
Complete response (CR) rate in (**a**) acute and (**b**) delayed phases

The CR rate of the overall phase was 73.1 and 74.1% in the control and aprepitant groups, respectively and there was no statistically significant difference (*P* = 1.00, Fig. 2). The CR rate in the acute and delayed phase are also shown in Fig. 3. The CR rate of the acute phase was 98.1 and 100% in the control and aprepitant groups, respectively and the corresponding values of the delayed phase were 75.0 and 74.1%, respectively. Furthermore, there were no statistically significant differences between the groups (*P* = 0.49 and 1.00, respectively). In the control group, 26.9% of the patients were administered the rescue dose, whereas, 24.1% of those in the aprepitant group were administered the rescue dose, without significant differences (*P* = 0.82).

### Comparison of severity of nausea

Table 2 shows the comparison of the severity of nausea between the groups, and the results indicate that there was no statistically significant difference in the acute and delayed phases.

**Table 2:** Severity of nausea

	Control group (*n* = 52)			Aprepitant group (*n* = 54)			*P*-value
	Grade 0	Grade 1	Grade 2	Grade 0	Grade 1	Grade 2	
Acute phase	51	1	0	54	0	0	0.32
Delayed phase	39	9	4	40	8	6	0.85

### Comparison of incidence of anorexia

**Fig. 4:**
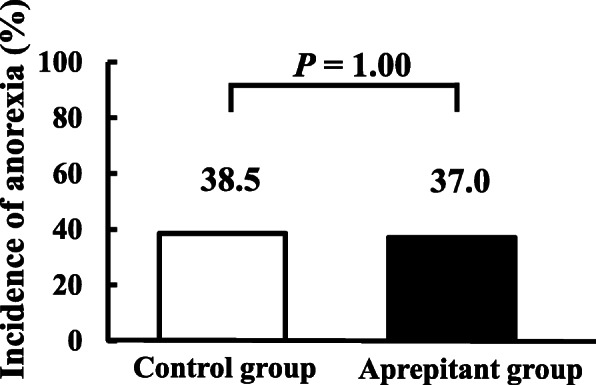
Incidence of anorexia

All anorexia appeared in the delayed phase, and its incidence was not statistically different between the control and aprepitant groups, at 38.5 and 37.0%, respectively (*P* = 1.00, Fig. 4).

## Discussion

Most of patients with gynecologic cancer are treated with a carboplatin-containing chemotherapy regimen [1–4]. CINV is one of the most serious side effects in cancer patients, therefore, its control is important to maintain patients’ quality of life and ensure continuation of chemotherapy [5–7]**.** Previous studies have recommended the addition of aprepitant to carboplatin-containing chemotherapy [5, 6, 13–18, 22, 23]. In particular, because female sex is a known risk factor for CINV [18], it would be important to evaluate its efficacy in gynecologic cancer patients. Therefore, we evaluated the efficacy of prophylactic administration of aprepitant, palonosetron, and DEX in carboplatin-based regimens in gynecologic cancer.

The results showed that the CR rate of the overall, acute, and delayed phases was not significantly different between the control and aprepitant group. Moreover, the severity of nausea and incidence of anorexia were not different between the groups. These results suggest that adding aprepitant did not attenuate CINV induced by carboplatin-containing regimens in gynecologic cancer.

In addition, medical care expenses for citizens have been increasing, and it is a concern with the use of aprepitant [24–26], therefore, its addition should be reconsider. Aprepitant is a high-priced drug, and the original branded drug cost 8949.3 Japanese yen (JPY), whereas generic drugs are 4511.2 JPY for a 3 day course. Fosaprepitant for intravenous infusion costs 13,978 JPY. The drug cost for a patient who receives six courses of a carboplatin-containing chemotherapy regimen, would be 53,695.8 JPY for the original bland name drug, 27,067.2 JPY for the generic agent, and 83,868 JPY for fosaprepitant. Carboplatin-containing regimens are generally administered as outpatient chemotherapy; thus, the drug cost directly affects the healthcare cost. As the use of additional rescue dose was similar between the groups, the medical cost in the aprepitant group might be higher than that in the control group. Further increases in the burden of health care expense are expected with the increase in cancer patients. From the viewpoint of health economy, aprepitant administration in carboplatin-containing chemotherapy regimens may need to be reconsidered.

Furthermore, the potential for interactions between aprepitant and other medications should be noted, because aprepitant is an inhibitor or inducer of CYP3A4 and an inducer of CYP2C9 [28–30]. Owing to the moderate inhibition of CYP3A4 by aprepitant, the metabolism of DEX, which is a CYP3A4 substrate, is decreased, leading to a 2.2-fold increase in the AUC of DEX and the guidelines recommend decreasing the dose of DEX by 50% when it is combined with aprepitant [28, 29]. In contrast, the metabolism of warfarin, which is a CYP2C9 substrate, is increased by aprepitant, resulting in a decrease in the international normalized ratio (INR); therefore, frequent INR monitoring is recommended for 2–3 weeks [30]. Because cancer patients often take multiple medicines, drug-drug interaction is one of the treatment challenges. Moreover, there is a risk of the patient forgetting to take aprepitant and infusion-site reactions caused by fosaprepitant [31].

Sugimori et al. have reported that the CR rate in the delayed phase was higher in the aprepitant group than in the control group, suggesting the usability of aprepitant [32]. A difference between our study and previous study is the DEX dosage on day 1. In the previous study, antiemetic therapy on day 1 was 13.2 mg DEX in the control group and 6 mg DEX in the aprepitant group, whereas that in our study was 16.5 mg in both groups. It is known that paclitaxel induces a hypersensitivity reaction; therefore, high-dose DEX administration is necessary according to the package insert. We consider that high-dose DEX administration on day 1 counteracted the effect of aprepitant in this study. We consider that this difference is important and reflects the situation in real-world clinical setting.

In our study, we used palonosetron as 5-HT_3_RA, but based on the drug cost, the first-generation 5-HT_3_RAs are better than palonosetron in the HEC regimens [26]. Moreover, there are reports that granisetron produces an equivalent CR rate to that of palonosetron in the overall phase [5]. However, some studies have reported that palonosetron is more effective than the first-generation 5-HT_3_RA agents [15, 22, 23]. Furthermore, it has been reported that 1 day and 3 days DEX administration with 0.75 mg palonosetron on day 1 have comparable antiemetic efficacy [33]. The combination of antiemetic drugs should be selected after considering their antiemetic efficacy and drug cost.

There are some limitations to the current study. First, this study was a retrospective review with a relatively small population. We were not able to evaluate the presence of motion sickness in baseline characteristics. Therefore, it would be necessary to perform a large-scale randomized prospective study to verify these results. Second, patients in this study were all female with gynecologic cancer and it would be better to make comparisons in a well-balanced population. Third, we evaluated the efficacy of aprepitant in the first course because several factors may affect CINV in the subsequent courses. Evaluation in multiple courses of chemotherapy may reveal other outcomes, thus warranting further research. Finally, we adopted a healthcare worker-based evaluation of CINV in this retrospective study; however, a subjective assessment by patients would provide a better evaluation of the antiemetic therapy.

## Conclusions

Our findings suggest that adding aprepitant to palonosetron and DEX does not prevent carboplatin-induced nausea and vomiting in gynecologic cancer patients. Therefore, adding aprepitant to palonosetron does not decrease carboplatin-induced nausea and vomiting in patients with gynecologic cancer.

## Data Availability

All data generated or analyzed in this study are included within the article.
